# Clozapine for Treatment-Resistant Disruptive Behaviors in Youths With Autism Spectrum Disorder Aged 10-17 Years: Protocol for an Open-Label Trial

**DOI:** 10.2196/58031

**Published:** 2025-01-30

**Authors:** André Luiz Schuh Teixeira da Rosa, Marina Ribeiro Barreto da Costa, Gabriela Bezerra Sorato, Felipe de Moura Manjabosco, Érica Bonganhi de Bem, Lucas Dellazari, Arthur Bezerra Falcão, Lucas de Oliveira Cia, Olivia Sorato Bezerra, Rogério Boff Borges, Luis Augusto Rohde, Ana Soledade Graeff-Martins

**Affiliations:** 1 Graduate Program of Psychiatry and Behavioral Sciences Department of Psychiatry Federal University of Rio Grande do Sul (UFRGS) Porto Alegre Brazil; 2 Division of Child and Adolescent Psychiatry Department of Psychiatry Federal University of Rio Grande do Sul (UFRGS) Porto Alegre Brazil; 3 Undergraduate Program of Medicine Federal University of Health Sciences of Porto Alegre (UFCSPA) Porto Alegre Brazil; 4 Child Neurology Unit, Department of Pediatrics Hospital de Clínicas de Porto Alegre (HCPA) Federal University of Rio Grande do Sul (UFRGS) Porto Alegre Brazil; 5 Department of Statistics Institute of Mathematics and Statistics Federal University of Rio Grande do Sul (UFRGS) Porto Alegre Brazil; 6 Unit of Biostatistics and Data Analysis Research Directorate Hospital de Clínicas de Porto Alegre (HCPA) Porto Alegre Brazil; 7 ADHD Outpatient Program & Developmental Psychiatry Program Hospital de Clinicas de Porto Alegre (HCPA) Federal University of Rio Grande do Sul (UFRGS) Porto Alegre Brazil; 8 Medical Council UNIFAJ Jaguariúna Brazil; 9 Medical Council UNIMAX Indaiatuba Brazil; 10 National Institute of Developmental Psychiatry & National Center for Innovation and Research in Mental Health Brasilia Brazil

**Keywords:** neurodevelopmental disorders, clozapine, psychopharmacology, antipsychotic medication, autism spectrum disorder, youth

## Abstract

**Background:**

Autism Spectrum Disorder (ASD) is a complex neurodevelopmental condition emerging in early childhood, characterized by core features such as sociocommunicative deficits and repetitive, rigid behaviors, interests, and activities. In addition to these, disruptive behaviors (DB), including aggression, self-injury, and severe tantrums, are frequently observed in pediatric patients with ASD. The atypical antipsychotics risperidone and aripiprazole, currently the only Food and Drug Administration–approved treatments for severe DB in patients with ASD, often encounter therapeutic failure or intolerance. Given this, exploring pharmacological alternatives for more effective management of DB associated with ASD is essential. Clozapine, noted for its unique antiaggressive effects in schizophrenia and in various treatment-resistant neuropsychiatric disorders, independent from its antipsychotic efficacy, remains underexplored in youths with ASD facing severe and persistent DB.

**Objective:**

This study aimed to evaluate the efficacy, tolerability, and safety of clozapine for treatment-resistant DB in youths with ASD.

**Methods:**

This is a prospective, single-center, noncontrolled, open-label trial. After a cross-titration phase, 31 patients with ASD aged 10-17 years and with treatment-resistant DB received a flexible dosage regimen of clozapine (up to 600 mg/day) for 12 weeks. Standardized instruments were applied before, during, and after the treatment, and rigorous clinical monitoring was performed weekly. The primary outcome was assessed using the Irritability Subscale of the Aberrant Behavior Checklist. Other efficacy measures include the Clinical Global Impression Severity and Improvement, the Swanson, Nolan, and Pelham questionnaire-IV, the Childhood Autism Rating Scale, and the Vineland Adaptive Behavior Scale. Safety and tolerability measures comprised adverse events, vital signs, electrocardiography, laboratory tests, physical measurements, and extrapyramidal symptoms with the Simpsons-Angus Scale. Statistical analysis will include chi-square tests with Monte Carlo simulation for categorical variables, paired *t* tests or Wilcoxon tests for continuous variables, and multivariate linear mixed models to evaluate the primary outcome, adjusting for confounders.

**Results:**

Recruitment commenced in February 2023. Data collection was concluded by April 2024, with analysis ongoing. This article presents the protocol of the initially planned study to provide a detailed methodological description. The results of this trial will be published in a future paper.

**Conclusions:**

The urgent need for effective pharmacological therapies in mitigating treatment-resistant DB in pediatric patients with ASD underscores the importance of this research. Our study represents the first open-label trial to explore the anti-aggressive effects of clozapine in this specific demographic, marking a pioneering step in clinical investigation. Adopting a pragmatic approach, this trial protocol aims to mirror real-world clinical settings, thereby enhancing the applicability and relevance of our findings. The preliminary nature of future results from this research has the potential to pave the way for more robust studies and emphasize the need for continued innovation in ASD treatment.

**Trial Registration:**

Brazilian Clinical Trials Registry RBR-54j3726; https://ensaiosclinicos.gov.br/rg/RBR-54j3726

**International Registered Report Identifier (IRRID):**

DERR1-10.2196/58031

## Introduction

The autism spectrum disorder (ASD) comprises a continuum of neurodevelopmental disorders that manifest in early childhood [1], characterized by impaired communication and social interaction, restricted and repetitive behavioral patterns, and occasionally impaired cognition or verbal language [2]. In the United States, the prevalence of ASD in 8-year-old children is 27.6/1000 (1 in 36 children) and 3.8-fold higher in boys than girls [3]. The diagnosis of ASD is an integrative process that includes the collection of current and developmental histories, direct observation, and clinical evaluation. Various standardized instruments may also support and substantiate the diagnosis [1]. Patients with ASD frequently present comorbid psychiatric conditions including, but not limited to, obsessive-compulsive disorder, intellectual disability, oppositional defiant disorder, mood and anxiety disorders, sleep disturbances, eating disorders, and attention-deficit/hyperactivity disorder [4].

The management of ASD involves a multidisciplinary approach, using behavioral and educational strategies to improve the patient’s quality of life and promote independence. First-line behavioral treatments comprise applied behavior analysis, cognitive behavioral therapy, and social skills training [5]. While pharmacological treatments do not directly address the core symptoms of ASD, they are used to manage psychiatric comorbidities and severe disruptive behaviors (DB) associated with autism (ie, irritability, hetero-aggression, self-injury, tantrums, and severe oppositional defiant symptoms) [6]. DB are common and can be observed in up to 1/4 of children with ASD [7]. Thus, pharmacotherapy can be pivotal in protecting the physical well-being of both autistic individuals and their caregivers, mitigating social exclusion, and enhancing overall quality of life [8]. The atypical antipsychotics risperidone and aripiprazole are the only drugs approved by the United States Food and Drug Administration for the treatment of irritability associated with ASD, and they are commonly used to treat DB in this population [9]. However, many patients do not respond adequately: approximately 30% of patients fail to respond to risperidone and 50% to aripiprazole [10].

It is essential to highlight that there are other psychosocial therapeutic resources (eg, behavioral therapies) and pharmacological treatments (eg, antidepressants and mood stabilizers) for managing DB. However, some patients, with a pronounced treatment resistance to these approaches, require the use of antipsychotics for the management of more severe symptoms. For those who do not respond adequately to the antipsychotics commonly used in clinical practice, clozapine emerges as a “last-resort” therapeutic alternative, as these patients still demand alternatives.

Clozapine, the first atypical antipsychotic developed, is the treatment of choice for treatment-resistant early-onset schizophrenia (EOS) and is noted for its anti-aggressive effects across several mental disorders [11]. Observational studies have also demonstrated its benefits in reducing treatment-resistant DB in ASD [12]. For instance, Beherec et al [13] analyzed medical records of 6 patients with ASD with refractory DB receiving clozapine, noting a reduction in the number of days with aggressive episodes and concomitant antipsychotic dosing. In addition, Rothärmel et al [14] reported maintained long-term benefits and favorable tolerability in their initial 6-patient cohorts. They corroborated these findings with a replication study involving an additional sample of 13 patients who exhibited a significant reduction in DB (65.2%, *P*=.003), leading to improved quality of life.

Clozapine therapy requires caution and rigorous monitoring during treatment due to its potential health risks, which include severe neutropenia [15], myocarditis [16], and seizures [17]. However, for some children and adolescents with ASD, the balance of risks and benefits is favorable, particularly when intense and treatment-resistant aggression leads to significant impairments [18]. Although preliminary and exploratory studies showed positive results, no prospective interventional studies with objective measures investigated the efficacy and tolerability of clozapine for treatment-resistant DB in pediatric patients with ASD, highlighting the need to explore it as a potential treatment for this subset of patients.

In this sense, this study aims to evaluate the efficacy, tolerability, and safety of clozapine in treating patients with ASD aged 10 to 17 years with DB resistance or intolerance to conventional antipsychotics. In addition, the study evaluates changes in adaptive functioning among patients and the impact of clozapine on caregivers’ quality of life. Drawing on prior evidence regarding clozapine’s specific anti-aggressive effects, this study explores its potential as a viable treatment for intractable cases. Accordingly, the hypothesis is that clozapine will prove efficacious and tolerable, with a safety profile acceptable for this clinical population. This article outlines the study protocol, which was initially drafted before the study's completion. Recruitment began in February 2023, and data collection concluded in April 2024. This protocol is now published to provide a comprehensive methodological description. Trial results will be reported in a future publication.

## Methods

### Study Design

This open-label, noncontrolled trial administers clozapine to all participants. The rationale for this study design, instead of a randomized controlled investigation, was based on the logistical and ethical challenges inherent in conducting a study within a pediatric ASD subpopulation characterized by refractoriness and severe DB. The study’s framework facilitates direct observation of adverse events and potentially severe risks related to clozapine use in vulnerable patients. Independent evaluators carry out baseline and follow-up assessments.

### Participants

The estimated sample consists of 30 patients aged 10 to 17 years who have been diagnosed with ASD and exhibit treatment-resistant DB.

### Eligibility

#### Inclusion Criteria

This study includes patients of both sexes aged between 10 and 17 years with a confirmed diagnosis of ASD according to the *DSM-5* (*Diagnostic and Statistical Manual of Mental Disorders* [Fifth Edition]) [19] criteria. Also, they must present DB (eg, psychomotor agitation, angry outbursts, oppositional defiant behavior, property destruction, self-injury behavior, or hetero-aggression) that are not caused by a coexisting medical or psychiatric condition, which could act as a trigger for the onset of these symptoms. Individuals must exhibit severe symptoms, impairing different function domains, reflected by a score of 5 or higher (markedly ill) on the Clinical Global Impression-Severity (CGI-S) scale, specifically anchored to DB. Treatment resistance, as defined by the research team, refers to the continued presence of severe DB despite adequate treatment (therapeutic failure) or intolerance to at least 2 antipsychotic agents, with at least 1 being atypical.

#### Exclusion Criteria

Patients who present the following conditions are excluded: unstable clinical illness or condition preventing the use of clozapine (eg, refractory epilepsy and heart or hematological diseases); insufficient psychosocial resources to follow the study protocol strictly (eg, correct drug administration and weekly attendance to the research center for clinical evaluations and blood testing); previous failure to clozapine treatment due to inadequate response after sufficient duration and dosing (a minimum of 400 mg/day for 6 weeks) or due to intolerable adverse events, such as leukopenia or cardiotoxicity; or pregnant, breastfeeding, or fertile patients not using an adequate contraceptive method.

### Recruitment

Our protocol uses a convenience sampling strategy. The study is disclosed in services for university hospitals, integral health of childhood and adolescence, and specialized assistance services for patients with neurodevelopmental disorders through informative material prepared by our research team, inviting patients, who meet the criteria for our study, to participate. Furthermore, we contact child and adolescent psychiatry outpatient clinics, special education institutes and ASD support groups in the metropolitan region of Porto Alegre in the state of Rio Grande do Sul (Brazil), disseminating our research and asking them to refer patients who could benefit from the intervention. It is also disclosed using traditional media (television, newspaper, and radio) and the Hospital de Clínicas de Porto Alegre (HCPA) social media website and institutional email. Legal guardians who intend to participate in the study contact the research team by phone or email.

### Screening

Caregivers interested in participating are invited to a screening assessment with a child and adolescent psychiatrist experienced in ASD to determine the patient’s eligibility for the study. During the evaluation, the psychiatrists verify the ASD diagnosis according to *DSM-5* criteria [19] and with the support of the Childhood Autism Rating Scale, Brazilian version (CARS-BR) [20]. Clinical features on the presence of severe DB and psychopharmacological treatment history are assessed. The research team informs caregivers about the study’s objectives, procedures, potential benefits, and risks associated with clozapine therapy, including side effects, and the mandatory requirement for weekly blood tests. Selected patients undergo a baseline evaluation, which includes data collection, laboratory tests, an electrocardiogram, and a physical examination. The psychiatrist confirms cardiometabolic and hematological stability as part of this initial assessment.

### Measures of Evaluation

Trained evaluators apply the following questionnaires and instruments before, during, and after the intervention:

#### Baseline Sociodemographic and Clinical Data

Data on age, sex, ethnicity, education level, socioeconomic status, clinical, and therapeutical history (eg, comorbidities, previous interventions, gestational and neonatal history, neurodevelopmental milestones, ASD diagnosis and support level, characteristics of DB, family psychiatric history, and response to previous and ongoing pharmacological treatments) are collected at the beginning of the study.

#### The CARS-BR

This scale comprises 14 items that help diagnose ASD in children and differentiate it from other developmental disorders [20]. It is fast to apply, suitable for all ages, and has objective and quantifiable scores through direct observation. The CARS-BR is used before and during the intervention to evaluate the central symptoms of ASD.

#### Schedule for Affective Disorders and Schizophrenia for School-Age Children–Present and Lifetime Version, DSM-5 Update

This semistructured interview evaluates the presence and severity of psychiatric disorders in children and adolescents in the present and throughout life [21]. In this study, it is used to assess psychiatric comorbidities.

#### CGI-S and Clinical Global Impression-Improvement

The Clinical Global Impression Severity (CGI-S) scale evaluates the global severity of the patient at baseline through an increasing score (1 to 7) considering the last 7 days [22]. The patient must score ≥ 5 (markedly ill) to meet the inclusion criteria. Also, the Clinical Global Impression–Improvement (CGI-I) compares the level of improvement or impairment of the patient every week after starting the pharmacological treatment through a score ranging from 1 (very much improved since the initiation of treatment) to 7 (very much worse since the initiation of treatment).

#### Aberrant Behavior Checklist, Irritability Subscale

Irritability is 1 of the 5 Aberrant Behavior Checklist (ABC) subscales with 15 items; it is easy to use and reliable [23]. This subscale is widely used in clinical trials to evaluate the efficacy of pharmacological treatments for irritability and DB in ASD, assessing several DB and changes over time. The ABC, Irritability (ABC-I) score change following clozapine treatment is the primary outcome of this open-label trial.

#### The Swanson, Nolan, and Pelham Questionnaire, Version IV

This questionnaire has 26 items to evaluate inattention, hyperactivity, impulsivity, and oppositional defiant behavior and is a complementary instrument to diagnose attention-deficit/hyperactivity disorder [24]. Our protocol uses this tool for additional assessment of DB.

#### Vineland Adaptive Behavior Scales, Third Edition

The Vineland Adaptive Behavior Scale, Third Edition (VABS-3) is a semistructured interview to evaluate adaptive behaviors divided into domains, such as communication (receptive, expressive, and written), daily living skills (personal, domestic, and community), socialization (interpersonal relationships, play and leisure, and coping skills), motor skills (gross, fine, and advanced motor coordination), and maladaptive behavior (internalizing, externalizing, and critical items) [25]. In our protocol, this tool evaluates how clozapine treatment impacts patients’ adaptive behaviors.

#### Side Effects Questionnaire Based on the Ugvalg for Kliniske Undergelser Side Effect Rating Scale for Psychotropic Drugs

The Ugvalg for Kliniske Undergelser Side Effect Rating Scale for Psychotropic Drugs (UKU) scale is divided into 4 sections, with 48 items evaluating the physical, psychological, neurological, and autonomic side effects of psychotropic drugs [26]. Each item is scored from 0 to 3, reflecting the intensity of the side effects. The scale has been specifically adapted for this study to monitor clozapine’s side effects comprehensively.

#### Simpsons-Angus Scale for Extrapyramidal Side Effects

This widely used scale consists of 10 items that evaluate the presence and severity of extrapyramidal side effects [27]. Each item is rated on a scale from 0 to 4, and the total score is obtained by summing the items and dividing them by 10, with scores up to 0.3 considered within the normal range. The Simpsons-Angus Scale for Extrapyramidal Side Effects (SAS) is a standard tool for evaluating drug-induced movement disorders.

#### Europe Health Interview Surveys Quality of Life, Abbreviated Instrument

The Europe Health Interview Surveys Quality of Life, Abbreviated Instrument (EUROHIS-QOL 8-item) assesses the quality of life across 4 domains, with 2 items per domain: physical, psychological, environmental, and social [28]. This study uses it to evaluate the caregivers’ quality of life, thus monitoring the potential effects of clozapine treatment on this aspect.

### Intervention

Throughout the study, a team of child and adolescent psychiatrists skilled in managing clozapine undertakes weekly evaluations of patients at the research facility. Out of 2 different evaluators assesses each patient. The first evaluator conducts the baseline assessment, and the second evaluator conducts all subsequent evaluations while following the same patient throughout the study protocol. This includes monitoring the therapeutic response of clozapine and managing any associated adverse effects. In addition, evaluators receive training to apply the evaluation measures accurately. Patients with significant medical comorbidities undergo specialist physician evaluation to confirm clinical suitability for the study. Eligible patients are prescribed clozapine therapy through a flexible titration regimen. Dose adjustments are made gradually over weeks to achieve a personalized dose. Implementing CGI scales during the titration phase facilitates the determination of an individualized fixed dose for each patient. Once the dosing has been stabilized, the clozapine intervention spans 12 weeks, during which the dose should remain constant. However, if necessary, minor changes may be made to ensure optimal patient outcomes. Blood tests follows the schedule outlined in Table 1. In addition, physical evaluations, including measurements of blood pressure, heart rate, abdominal and pelvic circumferences, weight, and BMI, are carried out weekly.

**Table 1:** Study timeline and measures of evaluation. From the beginning of the intervention to a stable clozapine dose, the titration duration varies to determine the most appropriate therapeutic dose for each individual patient.

	Baseline	DP^a^	V1^b^	V2	V3	V4	V5	V6	V7	V8	V9	V10	V11	V12
Physical evaluation^c^	✓	✓	✓	✓	✓	✓	✓	✓	✓	✓	✓	✓	✓	✓
CBC^d^	✓	✓	✓	✓	✓	✓	✓	✓	✓	✓	✓	✓	✓	✓
Monitoring of severe adverse events	N/A^e^	✓	✓	✓	✓	✓	✓	✓	✓	✓	✓	✓	✓	✓
Adverse events^f^	N/A	✓	✓	N/A	N/A	✓	N/A	✓	N/A	N/A	✓	N/A	N/A	✓
CGI-S^g^	✓	✓	✓	N/A	N/A	N/A	N/A	✓	N/A	N/A	N/A	N/A	N/A	✓
CGI-I^h^	N/A	✓	✓	N/A	N/A	N/A	N/A	✓	N/A	N/A	N/A	N/A	N/A	✓
Metabolic panel^i^	✓	N/A	✓	N/A	N/A	N/A	N/A	✓	N/A	N/A	N/A	N/A	N/A	✓
ABC-I^j^, CARS-BR^k^, and SNAP-IV^l^	✓	N/A	✓	N/A	N/A	N/A	N/A	✓	N/A	N/A	N/A	N/A	N/A	✓
VABS-3^m^	✓	N/A	N/A	N/A	N/A	N/A	N/A	N/A	N/A	N/A	N/A	N/A	N/A	✓
EUROHIS-QOL 8-item^n^	✓	N/A	✓	N/A	N/A	N/A	N/A	✓	N/A	N/A	N/A	N/A	N/A	✓
ECG^o^	✓	N/A	✓	N/A	N/A	N/A	N/A	N/A	N/A	N/A	N/A	N/A	N/A	N/A
K-SADS-PL^p^	✓	N/A	N/A	N/A	N/A	N/A	N/A	N/A	N/A	N/A	N/A	N/A	N/A	N/A
AST^q^, ALT^r^	✓	N/A	N/A	N/A	N/A	N/A	N/A	N/A	N/A	N/A	N/A	N/A	N/A	N/A

^a^aDP: Dose progression with a flexible titration schedule to reach a stable clozapine dose for each patient.

^b^V: visit.

^c^Weight, height, BMI, blood pressure, heart rate, and abdominal and pelvic circumference.

^d^CBC: Complete blood count.

^e^N/A: not applicable.

^f^Application of the Simpson-Angus Scale for extrapyramidal side effects and side effects questionnaire based on the Ugvalg for Kliniske Undergelser side effect rating scale for psychotropic drugs.

^g^CGI-S: Clinical Global Impression-Severity.

^h^CGI-I: Clinical Global Impression--Improvement.

^i^Fasting blood glucose, total cholesterol, high- and low-density lipoprotein, and triglycerides.

^j^ABC-I: Aberrant Behavior Checklist, Irritability Subscale.

^k^CARS-BR: Childhood Autism Rating Scale–Brazilian version.

^l^SNAP-IV: Swanson, Nolan, and Pelham questionnaire, version IV.

^m^VABS-3: Vineland Adaptive Behavior Scales-third edition.

^n^EUROHIS-QOL8: Europe Health Interview Surveys Quality of Life Abbreviated Instrument.

^o^ECG: electrocardiogram.

^p^K-SADS-PL: Schedule for Affective Disorders and Schizophrenia for School-age Children, present and lifetime version.

^q^AST: aspartate aminotransferase.

^r^ALT: alanine aminotransferase.

The initial dose of clozapine is 12.5 mg or 25 mg, divided into once or twice-daily doses. The starting dose of clozapine will be determined using an initial dose reference of approximately 0.3 mg/kg, rounded to the nearest multiple of 6.25 mg, with a maximum initial dose not exceeding 25 mg daily. Dose titration is tailored to the patient’s response and tolerability, up to 600 mg daily. Clinical management of clozapine considers individual conditions and comorbidities. The therapeutic range for most patients is expected to be between 200 and 400 mg/day. After the clozapine dosage reaches 100 to 200 mg per day, gradual discontinuation of other antipsychotics commences using plateau cross-titration. While the study does not mandate clozapine monotherapy, it aims the reduction of other psychotropics, particularly antipsychotics.

#### Clinical Monitoring and Adverse Event Management

Patients and caregivers receive educational materials on clozapine treatment and psychoeducational guidance from psychiatrists. Also, they can contact the researchers via phone in case of emergency symptoms (eg, fever, flu-like symptoms, sore throat, dyspnea, chest pain, and seizures). Caregivers are instructed to search for emergency services in case of severe symptomatology. Moreover, the team of psychiatrists is available to other physicians as required.

The study follows guidelines for clozapine treatment in EOS [29]. During all visits, psychiatrists proactively manage side effects to alleviate patients’ discomfort and optimize treatment adherence. If needed, side effects are treated symptomatically. Also, a consultant pediatrician and other specialists may be consulted whenever required. Advice on diet control and exercise is emphasized, and metformin may be used to mitigate weight gain and metabolic abnormalities from the beginning of treatment [30]. Prophylactic measures must be discussed with all caregivers to prevent constipation, and early prescription of stool softeners (ie, polyethylene glycol) might be used to avoid obstruction and ileus [31].

An electrocardiogram is performed before and after the clozapine treatment to assess the pharmacological impact on cardiac electric activity. Also, blood testing is performed before and during the treatment to monitor the risk of blood dyscrasias. To start clozapine therapy, patients must display the following hematological criteria: neutrophil >1500/μL and leukocyte >3000/μL [32]. Lithium carbonate may be prescribed to increase the neutrophil count in case of benign neutropenia [33], and the clozapine treatment must be suspended if neutrophil reduces under 1000/μL. Metabolic parameters (eg, fasting glucose, triglycerides, and cholesterol).

#### Data Collection and Management

Initial data is collected upon obtaining caregiver consent and confirming patient eligibility. Subsequently, qualified patients commence with clozapine therapy, marking the beginning of the treatment study and systematic clinical data acquisition. Measures of evaluation are applied as delineated in Table 1. Per the methodology of our study, we make provisions for remote consultations through video call using Google Meet, but only in exceptional circumstances in which patients cannot attend the research center personally. It is important to note that all participants must strictly adhere to the protocol of weekly blood sample collections, regardless of the consultation modality.

All evaluations are registered in HCPA’s electronic medical record system, and data is collected and managed using the Research Electronic Data Capture (REDCap) hosted at the HCPA. REDCap [34,35] is a secure web-based software to support data capture for research studies. It provides an intuitive interface for validated data capture, audit trails for tracking manipulations and data export, automated export procedures for continuous data downloads in common statistical packages, and procedures for data integration and interoperability with external sources.

#### Outcome Measures

The study will assess the efficacy of clozapine in reducing DB, as measured by ABC-I scores. Secondary measures include the CGI-S and Clinical Global Impression Improvement scales, ASD symptoms (CARS-BR), adaptive behaviors (Vineland Adaptive Behavior Scale - third edition), hyperactivity, impulsivity, and oppositional defiant behaviors (the Swanson, Nolan, and Pelham questionnaire), and quality of life of caregivers (EUROHIS-QOL 8-item). The safety and tolerability of the treatment are evaluated using an adverse effects inventory, the SAS, physical evaluation parameters, and blood testing (Table 1).

### Statistical Analysis

#### Overview

Qualitative variables will be presented as absolute and relative frequencies, and quantitative as mean and SD or median and IQR, as appropriate. The distribution of quantitative variables will be assessed using the histogram and quantile-quantile graph. Categorical variables will be compared using the chi-square test of independence with Monte Carlo simulation (when any cell in the contingency table presents an expected frequency < 5), and continuous variables before and after treatment will be compared using the paired *t* test or Wilcoxon signed rank test. The multivariate linear mixed model will assess the primary outcome to reduce possible confounding variables. The magnitude and direction of the association for quantitative secondary outcomes will be evaluated using the robust Poisson regression for binary variables and the generalized linear model for gamma-distributed variables. Multiple comparisons will be performed using the Bonferroni post hoc when needed. The significance level of 5% will be adopted for all analyses, which will be assessed using the PASW [Norman H. Nie] Statistics software (version 18.0 or higher) [36] and R [R Core Team] (version 4.3 or higher) [37].

#### Sample Size Calculation

The sample size was calculated to detect a reduction in the ABC-I score using the PSS Health tool (version 0.3.1) based on a relevant mean difference of 10 points [38]. Given a power of 99%, a significance level of 1%, and a SD of the expected difference of 9.7 [39], the calculation yielded a required sample size of 27 patients. Anticipating a 10% attrition due to losses and refusals, the sample size was adjusted to include 30 patients. Considering the vulnerable population and the demanding protocol, an over-enrollment strategy can be used to mitigate the impact of potential attrition further and ensure robustness.

### Ethical Considerations

This study fully complies with the Regulatory Standards and Guidelines for Research with Human Beings (Resolution 466/12) and upholds the ethical principles of the Declaration of Helsinki. Informed consent is obtained from legal guardians, who may choose to withdraw the patient from the study at any time without consequences. Whenever possible, patient assent is sought. In cases where it is not feasible, the legal guardian decides on the patient’s behalf. The study has received approval from the Research Ethics Committee of Hospital de Clínicas de Porto Alegre (CAAE 54677821.0.0000.5327), and its protocol is registered in the Brazilian Registry of Clinical Trials (RBR-54j3726). The research team continuously supports the patients and their caregivers throughout the study, especially regarding the emergence of alert signs associated with clozapine use. The Brazilian pharmaceutical company Cristália supplied the clozapine (Pinazan) for the participants. However, the company does not influence the study design, intervention, data analysis, or publication of the results. This ensures that the research team can make independent decisions.

## Results

Recruitment for the study commenced on February 10, 2023. A total of 33 patients were initially enrolled in the study. However, before the commencement of the intervention, 2 patients were withdrawn from the study due to their guardians’ decision not to participate further, and 3 patients discontinued treatment due to adverse events. Data collection was completed by April 2024. No primary or secondary outcomes are being reported in this manuscript, as it focuses solely on describing the study design and methods. The results of the study are expected to be published by June 2025.

## Discussion

It is anticipated that clozapine will demonstrate efficacy in reducing DB resistant to conventional treatments in youths with ASD. In addition, we expect to observe an acceptable safety profile by using rigorous monitoring in a pragmatic outpatient setting. In our view, this is the first quasi-experimental study to investigate the use of clozapine for this specific purpose and population. We foresee that our findings will align with several observational studies that have also highlighted clozapine as a valuable tool in similar cases.

Clozapine is recognized as a potential therapeutic alternative for addressing refractory aggressive behaviors in various neuropsychiatric disorders [12]. This study will explore the possibility that clozapine use may also extend to alleviating severe DB in children and adolescents with ASD. Regarding clozapine use to reduce aggression in young patients, a prospective open-label study showed a significant reduction of aggressive behavior in children and adolescents with treatment-resistant EOS during clozapine treatment for 12 to 24 weeks, reducing the need for emergency drugs and isolation [40]. In addition, observational studies suggested the efficacy of clozapine in patients with ASD and severe DB not responsive to conventional treatments [11,13,14]. However, only a small-scale, prospective intervention study explored the potential of clozapine in 3 children with ASD. In this study, Zuddas et al [41] evaluated 2 boys (8 years old) and 1 girl (12 years old) with DB resistant to haloperidol before and after the clozapine treatment. All children improved over 3 months, with a reduction in the Children’s Psychiatric Rating Scale scores from 99 to 60, 99 to 80, and 81 to 53. Although the boys demonstrated gradual improvement until 8-month follow-up (from 60 to 50 and 80 to 53), the girl had a complete relapse of symptoms after 5 months, with similar scores to baseline [41].

Considering that other studies examining the role of clozapine in ASD with treatment-resistant DB have maintained an observational design, prospective clinical studies with objective metrics and larger samples are needed to evaluate its efficacy. Thus, the present study aims to investigate the efficacy of clozapine in mitigating treatment-resistant DB in pediatric patients with ASD. The treatment of DB in ASD may improve adaptive behavior, which could favor the development of socialization, communication, cognition, daily activities, and the quality of life of patients and caregivers [8,42]. Also, behavioral improvement and clinical stabilization could allow patients to engage in other therapeutic modalities or actively participate in community activities or programs.

This study’s assessment of clozapine safety and tolerability in the pediatric ASD population is essential, particularly as these factors have been understudied in this group, unlike in patients with EOS [43]. A recent naturalistic study on clozapine in youth with neurodevelopmental disorders (ASD and/or ID) and treatment-resistant aggression showed significant improvements in clinical severity, functioning, and aberrant behaviors. Of the 26 patients, 61.5% were responders. Common side effects included increased appetite, sialorrhea, and repetitive behaviors, with 2 cases of seizures. This study supports clozapine’s potential in managing aggression in this population [44]. In contrast to the recently published study, our approach adopts a more interventional design. We also emphasize early detection and management of clozapine-induced side effects and serious adverse events. This includes intensive clinical monitoring through weekly evaluations by child and adolescent psychiatry specialists, pediatric support, regular laboratory tests, and close collaboration between doctors and caregivers. Furthermore, our study will evaluate the acceptability of clozapine outpatient treatment in this population, with a particular focus on adherence, considering the drug’s complex side effect profile, the frequency of required medical visits and blood tests, and the level of caregiver involvement. This approach aims to assess a practical framework for real-world clinical practice.

Regarding the limitations, the study features a small sample size and an uncontrolled open-label design, which introduces the possibility of biases, such as expectancy bias from both families and investigators. Nevertheless, this study constitutes an important initial step in clinical investigation, laying the groundwork for future controlled clinical trials with larger sample sizes. Furthermore, follow-up studies are necessary to evaluate the long-term efficacy and safety of clozapine.

Our dissemination plan involves publishing the results of this open-label trial in a high-impact international scientific journal, with the aim of providing evidence for the potential efficacy and safety of clozapine in managing DB in youths with ASD. Following this open-label clinical trial, our research team will conduct a longitudinal follow-up study with the same cohort to assess the sustained efficacy and safety of clozapine. In addition, a qualitative study is proposed to examine the caregivers’ experiences with clozapine therapy. Therefore, this trial is envisaged as a foundational step informing subsequent research endeavors.

## Abbreviations

ABC: Aberrant Behavior Checklist
ABC-I: Aberrant Behavior Checklist, Irritability Subscale
ASD: autism spectrum disorder
CARS-BR: CARS-BR: Childhood Autism Rating Scale, Brazilian version
CGI-I: Clinical Global Impression-Improvement
CGI-S: Clinical Global Impression-Severity
DB: disruptive behavior
DSM-5: Diagnostic and Statistical Manual of Mental Disorders (Fifth Edition)
EOS: early-onset schizophrenia
EUROHIS-QOL8: Europe Health Interview Surveys Quality of Life, Abbreviated Instrument
HCPA: Hospital de Clínicas de Porto Alegre
REDCap: Research Electronic Data Capture
SAS: Simpsons-Angus Scale
UKU: Ugvalg for Kliniske Undergelser Side Effect Rating Scale for Psychotropic Drugs
VABS-3: Vineland Adaptive Behavior Scale - third edition

## Data Availability

The dataset collected and examined in this study will be accessible through the lead author upon adequate request.
